# Mapping new theoretical and methodological terrain for knowledge translation: contributions from critical realism and the arts

**DOI:** 10.1186/1748-5908-4-1

**Published:** 2009-01-05

**Authors:** Pia C Kontos, Blake D Poland

**Affiliations:** 1 Toronto Rehabilitation Institute, 11035-550 University Avenue, Toronto, Ontario M5G 2A2, Canada; 2 Dalla Lana School of Public Health, University of Toronto, Health Sciences Building, 155 College Street, Toronto, Ontario M5T 3M7, Canada

## Abstract

**Background:**

Clinical practice guidelines have been a popular tool for the improvement of health care through the implementation of evidence from systematic research. Yet, it is increasingly clear that knowledge alone is insufficient to change practice. The social, cultural, and material contexts within which practice occurs may invite or reject innovation, complement or inhibit the activities required for success, and sustain or alter adherence to entrenched practices. However, knowledge translation (KT) models are limited in providing insight about how and why contextual contingencies interact, the causal mechanisms linking structural aspects of context and individual agency, and how these mechanisms influence KT. Another limitation of KT models is the neglect of methods to engage potential adopters of the innovation in critical reflection about aspects of context that influence practice, the relevance and meaning of innovation in the context of practice, and the identification of strategies for bringing about meaningful change.

**Discussion:**

This paper presents a KT model, the Critical Realism and the Arts Research Utilization Model (CRARUM), that combines critical realism and arts-based methodologies. Critical realism facilitates understanding of clinical settings by providing insight into the interrelationship between its structures and potentials, and individual action. The arts nurture empathy, and can foster reflection on the ways in which contextual factors influence and shape clinical practice, and how they may facilitate or impede change. The combination of critical realism and the arts within the CRARUM model promotes the successful embedding of interventions, and greater impact and sustainability.

**Conclusion:**

CRARUM has the potential to strengthen the science of implementation research by addressing the complexities of practice settings, and engaging potential adopters to critically reflect on existing and proposed practices and strategies for sustaining change.

## Background

In recent years, knowledge translation (KT) and evidence-based medicine have gained currency in health research through emphasis on moving 'knowledge off the shelves and into practice, making it relevant and accessible to practitioners and patients' [1]. Clinical practice guidelines have been a popular tool for the implementation of best clinical evidence from systematic research to improve the quality of health care. However, it is now widely understood that guidelines do not automatically change practice simply by establishing a knowledge base for practitioners [2]. Viewing clinical practice as 'an activity that simply attaches research to a local worksite' [3] overlooks the profound differences between settings in resources, as well as the established routines and cultural practices that influence and shape care [4].

Contrary to the view that best evidence can be disseminated across time and place and can achieve planned clinical behaviour change with reasonably predictable outcomes, a number of KT models have been developed to address the multiple and interrelated contextual interests, infrastructures, and procedures that are implicated in the adaptation of research to local health care practices [5,6]. Common to these models is attention to identifying, describing, and assessing the practice environment and its influences, which may facilitate and/or impede the process of research transfer and use [6-9]. Other common features of the KT models are monitoring the progress of the transfer effort, and evaluating usage of the evidence-based innovation and its impact on outcomes of interest [2,6,9].

Notwithstanding these significant strengths, many of the existing KT models suffer from particular oversights. First, while they assert an interconnection between the elements of the process of research utilization, most commonly there is no theory embedded within the models to explicate how these elements are interconnected, or how these interconnections facilitate or impede research transfer and use. Despite notable consensus that the use of theory is crucial in the design and evaluation of implementation research [2,6,10-12], it is rarely and often ineffectively used [11]. Critics suggest that theory development and use in the KT literature is a linear and discrete process [10,11] rendering implementation models ill-equipped to illuminate the complex interrelationships between various elements of the process of research utilization, including power relations, and how these interconnections facilitate or impede research transfer and use [2,13]. A second oversight is that for the most part only quantitative methods are endorsed for the evaluation of the use of the evidence-based innovation and its impact on outcomes of interest [9]. Pawson and Tilley [14] argue that reliance on 'hard' outcome measures alone in evaluation frameworks does not facilitate understanding of the complexity of organizational systems and the multiple realities of stakeholders. This suggests that there is a need for a pluralist approach to the evaluation of implementation research in order to understand the interactions and complexities involved in KT initiatives [2,8,15].

A third oversight of KT models is that where effective translation strategies are identified [16-18], arts-based methodologies are neglected despite their educational potential to foster critical awareness, encourage adopters to envision new possibilities, and affect change. Complex social interventions that target cognitive and/or psychosocial behaviour change are particularly difficult [14] because there is considerable leeway for misinterpretation, resistance, or even rejection of the innovation [19]. Therefore, it is imperative that complex interventions make use of approaches that facilitate critical self-reflection by professionals about how contextual and cultural factors influence and shape their understandings, assumptions and practices [8,20]. For the most part, however, KT strategies do not facilitate this kind of critical reflection; a limitation that is increasingly recognized [20,21].

**Figure 1:**
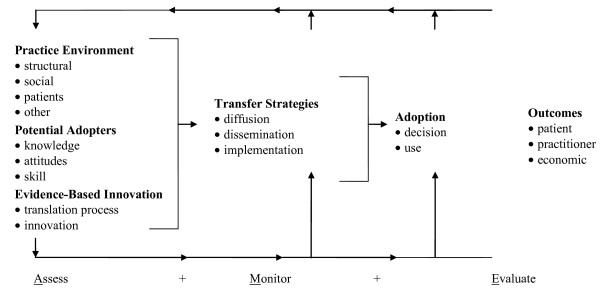
**The Ottawa Model of Research Use (OMRU)**. Logan, J, Graham, ID. Toward a comprehensive interdisciplinary model of health care research use. Science Communication 20/2: 227–246, copyright 1998 by Sage Publications Inc., Reprinted by Permission of Sage Publications Inc.

**Figure 2:**
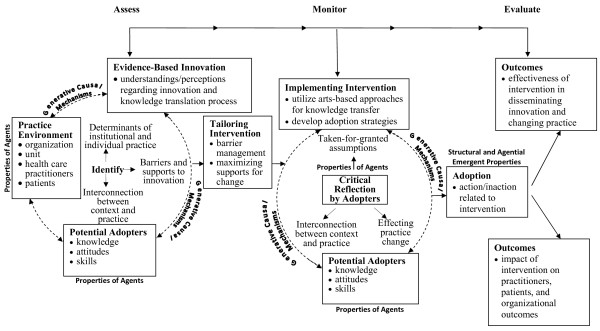
**Critical Realism & the Arts Research Utilization Model (CRARUM)**.

In seeking to transcend these oversights without forsaking the strengths of existing KT models, we advocate the integration of critical realism and arts-based methodologies into KT models that can best inform implementation research in the context of health care settings [12]. Such integration would: address the complexities of practice as a meaning-making activity; optimize interventions for local circumstances; target crucial factors in the organizational context that influence behaviour; disseminate evidence in a way that captures the imagination of practitioners and engages them in critical thought; and facilitate the achievement of best practice in health care settings. To illustrate this integration we have chosen the Ottawa Model of Research Use (OMRU, see Figure 1) [9], an adjuvant model [12] that is widely known and utilized [22] to promote the use and application of research in a variety of clinical areas such as neonatal intensive care [23], tertiary hospital care [24], ulcer care [25], and nurse call centres [26]. In order to distinguish our integration of critical realism and arts-based methodologies from the original OMRU, we have named our proposed model Critical Realism and the Arts Research Utilization Model (CRARUM, see Figure 2).

CRARUM shares a basic premise with OMRU, namely, that bridging the gap between research and practice is best achieved through the optimization of intervention and adoption strategies. As with OMRU, this is accomplished through the identification of factors and processes in the practice environment that promote and/or impede the adoption of research, and the setting-specific modifications of barriers and supports. Integral to this goal is the systematic process of assessing, monitoring, and evaluating the following six elements of research utilization: the practice environment; potential adopters; evidence-based innovation; strategies for transferring evidence into practice; evidence use; and outcomes of the process. Where CRARUM departs from OMRU is in its introduction of critical realism [27,28] into the model of KT. Specifically, it applies key concepts from critical realism such as structural and agential powers, and generative mechanisms [27,28] to more fully illuminate the processes of assessment, monitoring, and evaluation. The use of critical realism enables CRARUM to more accurately identify how the structural, agential, and intervention elements of the research utilization process are interconnected, and how these elements facilitate or impede action or inaction related to research uptake. Critical realism is a philosophical approach [27,28], central to which is the ontological claim that there is a dimension of reality that extends beyond observable phenomena, independent of individual perception, that includes deep underlying generative mechanisms that may or may not be triggered depending on circumstance. These mechanisms are real in the sense that they impact human activity, and thus must be accounted for when seeking to explain social phenomena. Yet their impact can only be tendential because of human reflexive abilities to resist or to strategically circumvent structural and social impingements [29]. Thus, the effect of generative mechanisms is contingent upon the reflexive deliberations and creativity of social agents. As such, critical realism is a perspective that can illuminate mechanisms embedded in clinical settings and interventions, and facilitate understanding of the outcomes that may or may not result, depending on whether and how the mechanisms are triggered, blocked, or modified by structural and agential capabilities. Here, critical realism is utilized as a theoretical base that informs the choice and development of interventions as well as the interpretation of implementation study results.

Additionally, as illustrated in Figure 2, CRARUM introduces the use of arts-based approaches for the translation of research evidence. Arts-based approaches are advocated for their potential to foster critical awareness about taken-for-granted assumptions, and the relationship between context and practice [30,31]. The arts elicits critical reflection by agents on the extent to which contextual/cultural factors influence and shape their understandings, assumptions, and practices, as well as how these factors facilitate or impede change efforts. As such, the use of the arts as a key KT feature can further facilitate tangible and lasting practice change.

## Discussion

### Unpacking the influence of context: a critical realist approach

Research utilization scholars have identified organizational context and culture as important factors influencing research use [2,12,32,33]. Kitson *et al. *[34], Estabrooks [35], and Lomas *et al. *[36] have persuasively argued that changing practice is not just a matter of focusing on the behaviour of individual practitioners but also requires attention to the social, cultural, and material context within which practice occurs. Contextual factors that have been identified as promoting the successful implementation of evidence into practice can be grouped under the broad themes of culture [37,38] and leadership [8,37,39]. Culture refers to the basic assumptions, values, and beliefs that are embedded in institutions and organizations [37,40]. Organizations most conducive to facilitating change are those that are described as 'learning organizations' [37,41], which refers to an organization's capacity to recognize the value of new knowledge, assimilate it, and then implement it as the basis of decision-making. As part of such a context, decentralized decision-making, collaboration, teamwork, receptivity to change, and shared goals for improvement are typically valued [8,37,38,40]. Reducing the uncertainty that results from inconsistencies in unit management practices, technology-driven routine work, and the complexity of teamwork have also been identified as necessary precursors to increasing research utilization [32]. The chances of successful implementation are enhanced further in contexts where clinical decision-making is informed not only by evidence from systematic reviews and randomized controlled trials, but also by patient preferences and clinical experience [37,42]. It is argued that there is greater likelihood for successful implementation where evidence is consistent with patient narratives and experiences as well as the tacit knowledge of practitioners [42]. Considering clinical experience and patient preferences as valid sources of evidence requires broader evaluative techniques than 'hard' outcome measures alone [8]. Thus organizations most conducive to research use have the resources to incorporate multiple methods and sources of feedback into their evaluative framework [42].

McCormack *et al. *[8] have emphasized the importance of transformational leadership, also referred to as shared partnership and distributed leadership [39], for creating a culture of inclusion that values all levels and rank of staff. Balanced power, shared purposes and goals, shared responsibility for work, and mutual respect are requirements of shared leadership [39] to effectively alter the prevailing organizational culture and to create a context more conducive to the integration of evidence and practice.

Clearly, contexts can invite or reject innovation, complement or inhibit the activities required for success, and sustain or alter adherence to entrenched practices [39]. While there has been much progress by way of identifying which aspects of context may influence innovation adoption in healthcare, much less progress has been made in terms of understanding how and why contextual contingencies interact the way they do, and how these interactions influence KT [32]. This is troubling, given the importance of understanding context for facilitating successful implementation. Many conceptual models depict relationships between the various different aspects of context [9,37,43] without recourse to theory to facilitate understanding of why relationships assume the form they do, and the underlying factors of the complex realities of practice. Even those models that are informed by theory are limited in their capacity to conceptualize causal mechanisms that link structure and agency. Either there exists such deep bias in favour of viewing structural properties as overconditioning the actions of agents (*e.g.*, situated-change theory [44] and theories of culture [45]), or quite the reverse, agency is said to be the primary characteristic or driving force of behaviour (*e.g.*, planned behaviour [46] and community of practice [47] theories).

Critical realism, with its commitment to elucidating both the structures which constrain and enable activities, and how individual action reinforces, challenges, or transforms structural impingements, offers a promising way to remedy the tendency to either strip agency of structure or structure of agency. Critical realism has effectively been used to evaluate cardiac rehabilitation programmes [48] and diagnostic and treatment delays in breast cancer [49]. It has informed an analysis of the effects of racism on occupational relationships between nurses and doctors, and how these are mediated by professional ideology [50], the sociopolitics of evidence-based medicine [51], as well as the fields of evaluation (realist evaluation) [14], organizations [52] (see the notion of engaged scholarship [53]), and health promotion [54].

Critical realism furnishes a sophisticated understanding of context. In critical realism [27,28], a distinction is made between the real (underlying nature and causal powers of objects/agents), the actual (what happens if/when those powers are activated), and the empirical (what is experienced/observed) [55,56]. This distinction is central to an ontological conviction that there exists a reality distinct from and greater than the domain of the empirical [27,28], and that this reality is comprised of structures and mechanisms independent of our perceptions. Mechanisms can coincide under real world conditions to produce emergent properties contingent in time and space, properties which are irreducible to those of their constituents [55]. The notion of contingency contrasts with positivist notions of universal logical necessity (natural laws, generalizable truths) by highlighting the uncertain nature of phenomena (*i.e.*, that propositions may hold true only under certain circumstances). In the domain of the actual, there are many mechanisms concurrently active where some reinforce one another, and others frustrate the manifestations of each other. In this sense, it can only be said that a certain object tends to act or behave in a certain way [57].

Danermark *et al. *[57] use the example of a match to illustrate the notion of tendency. The object (match) has within it the causal power for fire, but ignition requires this power to be triggered by agential mechanisms through the act of striking, as well as by mechanisms of nature including sufficient oxygen, dry conditions, etc. Irrespective of an agent's intent, numerous combinations of mechanisms may influence whether the causal power (fire) will manifest itself in the realm of the empirical. Thus generative mechanisms are real in the sense that they provide the conditions that serve to constrain or enable an individual's action. For critical realists, explanatory power derives not from counting the co-presence of observable phenomena and inferring causation on the basis of empirical co-occurrence, but from 'identifying causal mechanisms and how they work, and discovering if they have been activated and under what conditions' [58]. Consequently, context becomes redefined as the interrelationship between real and emergent or possible properties of structures and agents:

The (local) mix of conditions and events (social agents, objects, and interactions) that characterize open systems, and whose unique confluence in time and space selectively activates (triggers, blocks, or modifies) causal powers (mechanisms) in a chain of reactions that may result in very different outcomes depending on the dynamic interplay of conditions and mechanisms over time and space [54].

In illuminating these aspects of context, critical realism is a perspective that is equally pertinent to program evaluation. Proponents of critical realist evaluation [59,60] have argued that the central question is not so much whether certain interventions work in a generalizable way, but what will work with these stakeholders/actors in this setting at this time. This shifts the focus of evaluation of interventions from a programme-based view of what works to causal pathways [51]. Opening the 'black box' [61] of implementation is necessary to better understand the relations between the innovation and structural and agential properties [62] that inform uptake, the need for refinement, and the factors important for replication [63].

For critical realists, agential capacity is not innate or static, but relational. It is activated in the mobilization of various forms of capital: social, cultural, and material/economic [64]. Power is exercised in relation to others who are likely to mobilize stocks of capital and resources in order to promote their own interests. Human action is enabled and/or constrained by power inequities, but this action, in turn, reproduces and/or transforms those structures of power [49]. For example, those perceived as having specialized knowledge, and social and economic authority often prevail. These stocks of capital are not randomly assigned but tend to follow time-honoured cleavages of race, gender, and social class, suggesting that social structures (including institutional practices, policies and regulations, cultural norms) play a role in the production and often (re)production of inequalities amongst social groups. This 'indebtedness of agency to structure', as Scambler [65] terms it, underscores the dialectical relationship that exists between human action and structures of power [49].

Power relations may be ubiquitous, but they are expressed in different ways in different settings, in part because other mechanisms are also at play which may be local manifestations of much broader processes (*e.g.*, gender and race relations, management-labour relations). Contemporary neoliberal 'logics' of management practice (concerned primarily with profitability, cost reduction, cost-per case efficiency, and standardization) must figure prominently in any such discussion. This kind of managerialism [66] seeks to parse healthcare into discrete tasks that can be easily measured against written standards pertaining to how much time can be spent on a given task and how it should be done [67]. The measure of care lies with the physical task rather than the quality of human interaction and, as a consequence, the relationship between the care provider and recipient is not always quintessentially one of caring unless those most closely involved make a point [68] and have the requisite capital [49], to make it so.

Thus interventions aimed at 'humanizing' care must acknowledge that such interventions intersect powerfully with other dynamics (decision latitude, service delivery trends, atomization of the nuclear family leading to loss of proximal family members, etc.) in ways that, by virtue of the underlying causal powers at play, have the ability to either enhance or undermine change initiatives. Critical realism proffers a view of evidence-based practice that concentrates on an elaboration of mechanisms and the logic of causation rather than a programme-based view of what works in terms of research-manipulated interventions and independent outcome measures. It is an approach to implementation evaluation (also referred to as formative evaluation) [63] that, when combined with outcomes evaluation, creates a powerful 'hybrid style approach for implementation research' [63] which provides a clearer direction for action because the decision maker not only has knowledge of the outcomes but also of what produced the occurrence or absence of targeted outcomes.

### Recovering agency: an agenda for active engagement

More is at stake here than an exhortation to be mindful of context as a kind of general backdrop for interventions. In seeking to understand how mechanisms play out in a particular setting, with particular agents at a specific time, we must also take account of how reflexive agents perceive, negotiate, unwittingly reinforce or selectively resist the effects of these broader trends and influences in the context of their own life biographies, socialization, and the micro-social context of peer relations in the workplace. Critical realism is a perspective that deems the creative tactics of individuals to deal with impingements in the social and material contexts of everyday life to be of equal importance to the social structures that furnish such impingements [49]. Deep underlying generative mechanisms do form the basis for structural impingements on human activity, but structural relations of gender, class, and race can for example be actively resisted or reproduced during encounters with the healthcare system by practitioners (or patients) mobilizing their own stocks of capital in particular settings and contexts [49].

Diverse disciplines, practices, and literatures have identified the problematic nature of engagement as a central issue for a myriad of professional practices. Taking agency seriously means finding ways to work with practitioners to help them understand their situation, examine their values, identify barriers and opportunities for change, implement solutions, and evaluate the results while never losing sight of the ways in which generative mechanisms operate to constrain and/or enable change in particular settings. This requires a more sophisticated approach to engagement and dialogue that draws in and works with the whole person in his or her 'multiple literacies' [69]. This is where the arts, as a medium for reaching and engaging care providers, can be particularly powerful.

### Staging the data for research transfer

There can be considerable leeway for evidence to be (mis)interpreted, resisted, adapted, and even dismissed by potential adopters [19]. It is therefore imperative that when bringing evidence-based innovations to practice and encouraging their adoption, use is made of approaches that view potential adopters as beings capable of reflecting critically on their own assumptions, and on the relationship between their practice and its context [15]. KT strategies have ranged, for the most part, from passive unplanned efforts (diffusion; *e.g.*, publication of research findings), to targeting and tailoring the evidence and the message for a particular audience (dissemination; *e.g.*, direct mailing), to systematic efforts to encourage adoption of the evidence (implementation, *e.g.*, use of incentives and sanctions) [70]. There is evidence to suggest that interactive educational interventions such as workshops can result in significant changes in professional practice [17]. The arts, however, have been neglected as a KT strategy despite their enormous interactive, educational, and emancipatory potential; an omission that our model, CRARUM, specifically remedies.

Literature and theatrical performance are increasingly being used as a means to humanize medical education [71]. Shapiro and Hunt [72] contend that live theatrical performances contribute significantly to medical education because they have 'a uniquely compelling emotional quality, making it difficult to avoid or intellectualize the struggles and suffering portrayed.' A growing number of health researchers are turning to theatrical performance as an innovative approach to extending research findings beyond the discipline in which they were generated, and thereby making research more accessible and relevant in health care settings [30,73-75]. Examples of research-based productions include schizophrenia [76], substance abuse [77], breast cancer [73], prostate cancer [78], ovarian cancer [72], AIDS [72], Alzheimer's disease [30], and traumatic brain injury [79]. Dramatic performance is particularly effective in engaging imagination and fostering sympathy because it privileges the phenomenological complexity of life. It draws the observer into a particular social and cultural world with all its textures, sounds, gestures, and movements [30] in contrast to textualism, which flattens out 'the flux of human relationships, the ways meanings are created intersubjectively as well as intertextually, embodied in gestures as well as in words...' [80].

Dramatic performances have been successful in helping practitioners and medical trainees reflect on the care they provide and increase their understanding of patient care issues [72,78,81]. For example, in post-performance evaluations of the drama *No Big Deal? *[78], based on a study of prostate cancer survivors and their spouses, oncology physicians, nurses, and allied health professionals indicated that attending the performance resulted in a new level of awareness or understanding of how patients are affected by cancer diagnosis and treatment. Post-performance evaluations of a research-based drama about personhood in Alzheimer's disease [31] found experienced nursing and allied health professionals acquired a new level of understanding of the expressiveness of persons with cognitive impairment. Deloney and Graham [82] have similarly validated the use of drama as an effective method to provide training about end-of-life issues and doctor-patient communication. These evaluations support the effectiveness of research-based drama as a KT strategy with the potential to positively impact practice.

Improvisational theatre is an important form of drama that is influencing the way social and health scientists are incorporating drama into their research [83]. Developed out of the political-theatrical agenda of Augusto Boal, a Brazilian theater director, writer, and theorist, 'forum theatre' is a method of teaching lay non-actors how to recognize and transform the conditions of oppression in their lives. The theatrical goal is to engage the disempowered and to create ways to liberate the disenfranchised [84]. A short play is performed, followed by an identical presentation in which audience members are encouraged to rise and physically replace the main character when they feel inspired to enact an alternative approach that might result in a more favourable outcome [85]. Highly interactive and imaginative, forum theatre fosters critical thinking about the lived reality of the participants, the root causes and solutions to social problems, and change. The collaborative process is intended to address the need for participants to step outside 'the apparently solid 'matrix' of 'this time in this place' and collectively de-codify the 'myth of fixed reality' – engendering hope for transformation' [85]. Attitudes, beliefs, conflicts, failures, successes, and aspirations are shared, and emerging from this process is a vision of how things could be different [83]. Mienczakowski, for example, has used elements of Boal's forum theatre techniques in ethnographic performance projects about schizophrenia and alcoholism [83]. His use of these techniques was intended to provide emancipatory opportunities and insights for both health professionals and health consumers [83].

By offering the potential to foster critical awareness, to facilitate understanding, and nurture sympathy, arts-based approaches are well positioned to strengthen initiatives that seek to transform health care. In a recent review of the literature on the use of research-based drama for KT [86], a number of areas for further exploration were identified. First, little is known about the extent to which drama impacts health audiences, why it has the impact that it does, and whether and how this impact leads to real world application. Second, distinguishing the aesthetic qualities of the performance from its content has yet to be done, and this too would lead to a better understanding of the particularities of drama that work as a KT strategy. Finally, because the most common methods employed in evaluation studies have been unstructured feedback (*e.g.*, reflective journals from students and informal discussions), and self-report questionnaires, qualitative methods are recommended to generate a rich data set for understanding how research-based drama operates as a KT strategy.

### Moving from theory to practice

How would CRARUM help to guide users in successfully implementing evidence into health care settings? Following the logic of critical realism, as a necessary first step, qualitative and quantitative methods of data collection can serve to identify causal generative mechanisms of existing care. These data reveal contradictions between espoused and enacted practice, and existing barriers to best practice. Elucidating the social, cultural, and material conditions under which practice occurs enables the intervention to be meaningfully individualized to the care setting. For example, understanding the context of long-term care would be critical when implementing an educational intervention for front-line dementia care practitioners about a patient-centred approach to care. Understanding how administrators and practitioners negotiate the potential paradox of front-line staff being mandated to provide patient-centred care, despite healthcare rationalization policies that constrain their ability to do so, would further inform the development of adoption strategies for facilitating the use of the new approach to practice. Moreover, because organizational hierarchies can limit practice change [20,87], engaging administrators in the development of adoption strategies can increase the likelihood of successful implementation. Theorizing the dynamic interrelationship between individual agency, organizational rules and regulations, and the larger health care restructuring agenda can facilitate the tailoring of the intervention such that its relevance, feasibility, and success are maximized.

Yet, tailoring interventions to better fit local settings alone is insufficient to achieve optimal care settings. The arts provide an innovative approach to the challenge of engaging practitioners to imagine new possibilities for more humanistic caregiving practices by helping practitioners to meaningfully connect with their care recipients [31]. The use of drama, for example, can raise critical awareness of taken-for-granted assumptions about standard care practices, and affect change through reflection on the nexus of personal assumptions, staff behaviour, and organizational policy [88]. In so doing, it can facilitate the development and implementation of an agenda for change that derives from the critical awareness of stakeholders themselves [15].

Critical realist evaluation of an intervention would take into account both the process and context of change. This entails an exploration of outcomes (*e.g.*, non-pharmacological approach to behavioural management in dementia care), but also the conditions that were present to enable those outcomes (*e.g.*, administrator support of practitioners' adaptation of care to meet patient preferences, such as having an evening bath rather than a morning bath, instead of rigid enforcement of institutional routines). Qualitative and quantitative data collection can inform understandings of what did/did not occur within the setting relative to the intervention, and which structural and agential factors influenced adoption of, or resistance to the intervention. Thus, in addition to answering whether the intervention works, critical realist evaluation facilitates understanding of why it worked, for whom, and in what circumstances.

## Conclusion

KT, which is central to evidence-based medicine, has been identified as the most important contemporary initiative committed to reshaping biomedical reasoning and practice [3]. While the move to establish scientific research as a fundamental ground of medical decision-making has had an enthusiastic reception, it has also generated considerable debate [3,51,89]. Critics have focused on the separation that evidence-based medicine creates between research and practice-based settings and the one-way linear model of the relationship between the two that it creates [51]. Indeed, built into the evidence-based movement is the assumption that clinicians can take standardized guidelines and easily translate them into the 'messy' realities of clinical engagement [51]. It is our contention that KT initiatives that neglect the settings for practice change can undermine successful uptake, as well as prediction about what will work best in a given context. Another limitation of KT initiatives is their neglect of methods to engage potential adopters of the innovation in critical reflection about practice, the relevance and meaning of innovation in the context of their practice, and the identification of strategies for bringing about meaningful change in practice settings.

Given the inescapably interpretive dimension of evidence [19] and the complexity of health care settings [90,91], we advance a KT model, CRARUM, which we believe overcomes limitations of earlier models. We have incorporated critical realism in the model to shed light on the structures, powers, generative mechanisms, and tendencies that characterize clinical settings and the agential reflexive capabilities of health care practitioners. We have argued that these data will not only help successfully embed interventions in settings, thereby ensuring greater impact and sustainability, but also generate understanding of how and why interventions work (or fail) in a particular setting including the actual degree of adoption, and the extent to which the adoption occurred as intended [63]. Furthermore, in its emphasis on arts-based methodologies, CRARUM underscores the importance of engaging potential adopters as agents capable of reflecting critically on their own assumptions, and on the relationship between their practice and its context. Central to this critical reflection amongst practitioners is an examination of the relevance and feasibility of the evidence-based innovation in relation to other political, strategic, contextual, and stakeholder considerations.

Given the ascendancy of KT, CRARUM has the potential to make an important contribution to implementation research. Clegg makes a compelling argument for critical realism, with its underlying themes of critique and emancipation, in that it offers a distinctive approach to the debate about evidence-based practice [51]. We go further by combining critical realism and arts-based methodologies in a way that enables agency to take centre stage and to reclaim KT for critique and emancipation.

## Competing interests

The authors declare that they have no competing interests.

## Authors' contributions

PK and BP developed the CRARUM model. PK is the lead author and coordinator of the paper. BP was involved in drafting the paper and revising it for inclusion of critically important intellectual content. Both PK and BP read and approved the final draft of the paper.
